# Iodine-supported implants in prevention and treatment of surgical site infections for compromised hosts: a prospective study

**DOI:** 10.1186/s13018-023-03868-5

**Published:** 2023-05-27

**Authors:** Toshiharu Shirai, Hiroyuki Tsuchiya, Ryu Terauchi, Shinji Tsuchida, Seiji Shimomura, Yoshitomo Kajino, Kenji Takahashi

**Affiliations:** 1 Department of Orthopaedics, Graduate School of Medical Science, Kyoto Prefectural University of Medicine, 465 Kajii-cho, Kamigyo-Ku, Kyoto, 602-8566 Japan; 2 Department of Orthopaedic Surgery, Graduate School of Medical Science, Kanazawa University, 13-1 Takaramachi, Kanazawa, 920-8641 Japan

**Keywords:** Surgical site infection, Postoperative infection, Re-infection, Antibacterial, Iodine, Iodine-coated implant, Implant failure

## Abstract

**Background:**

Surgical site infection (SSI) is a common complication following orthopedic implantation. We developed an iodine coating for titanium implants to reduce implant-related infections and conducted a prospective clinical study to evaluate the efficacy and potential drawbacks of iodine-supported implants.

**Patients and methods:**

Between July 2008 and July 2017, 653 patients (377 male and 27 female patients; mean age, 48.6) with postoperative infection or a compromised status were treated using iodine-loaded titanium implants. The mean follow-up period was 41.7 months. In 477 patients, iodine-supported implants were used to prevent infection and in 176 patients, to treat active infection (one-stage surgery, 89 patients; two-stage surgery, 87 patients). In the limbs and pelvis, the primary diagnoses included the following: 161 tumors, 92 deformities/shortening, 47 pseudarthrosis, 42 fractures, 32 infected TKA, 25 osteoarthritis, 21 pyogenic arthritis, 20 infected THA, and 6 osteomyelitis. In the spinal cases, there were 136 cases of tumors, 36 cases of pyogenic spondylitis, and 35 cases of degeneration. Five modes of implant failure were identified and classified as follows: soft tissue failure (type 1), aseptic loosening (type 2), structural failure (type 3), infection (type 4), and tumor progression (type 5).

**Results:**

The overall failure rate in our series was 26.3% (172/653). There were 101 mechanical failures, including 22 type 1, 20 type 2, and 59 type 3 failures. Non-mechanical causes accounted for 71 failures, including 45 type 4 and 26 type 5 failures. The overall incidence of infections was 6.8%. The mean time to the onset of infection after implantation was 9.1 months. The overall infection rate was 3.7% in the prevention cases and 15.3% in the treatment cases. There was no difference between one-stage replacement (14.6%) and two-stage replacement (16.0%). There were 11 cases of treatment for SSI of spine surgery, and the re-infection rate was 0% using iodine-coated instruments.

**Conclusions:**

The five modes of failure of the iodine-supported implant were satisfactory compared with previous reports. In particular, because the infection rate of iodine-coated implants used for compromised hosts is low compared with other methods, postoperative infection is more easily controlled. It can be considered highly effective for spinal infections that require one-stage revision surgery.

*Level of evidence* IV.

*Trial registration* Prospective, Observation study.

## Introduction

The number of surgeries that involve the use of implants is increasing annually in Japan, which is becoming a super-aging society. Approximately 100,000 total knee arthroplasty (TKA) and 70,000 total hip arthroplasty (THA) have been performed, which is approximately twice the number performed 10 years ago. As a result, implant-related surgical site infections (SSI) are increasing. SSI is one of the most serious complications of orthopedic surgery. Many studies have been conducted on implants with antibacterial treatments to reduce surgical site infections. In particular, gentamicin-coated tibial intramedullary nails and silver-coated megaprostheses have been clinically applied and good results have been reported. However, there are concerns about the problem of resistant bacteria, short-term persistence in antibacterial processing, and toxicity such as algyria in silver coating. Therefore, we developed an iodine-coating method. Iodine has a wide antibacterial spectrum and is characterized by the absence of resistant bacteria. In addition, the biosafety of iodine has been established since it has been used as a medical agent as a disinfectant and contrast medium. Since 2005, we have conducted basic research on iodine coatings and have reported their usefulness [1, 2]. A prospective clinical study using iodine-coated titanium implants in compromised hosts and patients with postoperative infection, which was approved by the ethics committee of our institution, commenced in 2008. In this study, we evaluated the efficacy and potential problems of iodine-coated implants and reported the final results.

## Patients and methods

### Iodine coating

In this study, we developed an iodine coating for titanium implants. The anodic oxide film was produced electrically, and the use of a povidone-iodine electrolyte resulted in the formation of an adhesive porous anodic oxide with antiseptic properties of iodine. The thickness of the anodic oxide film containing iodine was between 5 and 10 mm, with more than 100,000 pores/mm^2^ and a capacity to support 10–12 mg/cm^2^ of iodine.

### Clinical study

This study was approved by the institutional review board of our university. Between July 2008 and July 2017, 653 patients with postoperative infection or a compromised status were treated using iodine-loaded titanium implants. The mean patient age was 48.6 years (range, 4–90 years). The mean follow-up duration was 41.7 months (range, 5–121.5 months). Three hundred seventy-seven patients were male and 276 were female. Iodine-supported implants were used to prevent infection In 477 patients, such as in immunocompromised patients, and to treat active infections in 176 patients (one-stage surgery, 89 patients; two-stage surgery, 87 patients). In the limbs and pelvis, the primary diagnoses included the following cases: tumors, 161; deformity/shortening, 92; pseudarthrosis, 47; fracture, 42; infected TKA, 32; osteoarthritis, 25; pyogenic arthritis, 21; infected THA, 20; and osteomyelitis, six. In the spinal cases, there were 136 cases of tumors, 36 cases of pyogenic spondylitis, and 35 cases of degeneration (Table 1).

**Table 1:** List of patient diseases

Disease	No. of cases	No. of prevention cases	No. of treatment cases
Extremity/Pelvis	446	325	121
Tumor	161	138	23
Deformity/shortening	92	90	2
Pseudarthrosis	47	28	19
Fracture	42	39	3
Infected TKA	32		32
OA (hip, knee, ankle)	25	23	2
Pyogenic arthritis	21	5	16
Infected THA	20		20
Osteomyelitis	6	2	4
Spine	207	152	55
Tumor	136	125	11
Pyogenic spondylitis	36		36
Degenerative spondylitis	35	27	8

*TKA* total knee arthroplasty, *OA* osteoarthritis, *THA* total hip arthroplasty

Implant failures, such as mechanical and non-mechanical failures, were classified by modifying the classification by Henderson et al. [3]. Mechanical failures included type 1 soft tissue failure, type 2 aseptic loosening, and type 3 structural failure (peri-implant or implant fracture). Non-mechanical failures were those requiring surgical removal or revision without primary loss of structural integrity of the implant: type 4, infection; type 5, tumor progression (recurrence or progression of tumor) [3]. In this study, it is important to show the percentage of infections (type 4 failure).

## Results

The overall failure rate in our series was 26.3% (172/653). There were 101 mechanical failures, including 22 type 1, 20 type 2, and 59 type 3 failures. Non-mechanical causes accounted for 71 failures, including 45 type 4 and 26 type 5 failures (Table 2).

**Table 2:** Types and incidence of complications according to Henderson et al. classification [3]

	No. of case	Relative incidence (%)	Absolute risk (%)
Mechanical	101	58.7	15.4
Non-mechanical	71	41.3	10.8
Type 1, Soft tissue failure	22	12.8	3.3
Type 2, Aseptic loosening	20	11.6	3
Type 3, Structural failure	59	34.3	9
Type 4, Infection	45	26.2	6.8
Type 5, Tumor progression	26	15.1	8.7

Soft tissue failure (type 1 failure) occurred in 22/653 cases (3.3%). Aseptic wound dehiscence was the most frequent type 1 failure and occurred in 12/22 of cases (54.5%); all cases were treated with debridement and drainage. Other type 1 failures included four dislocations of the hip (9%:4/43) treated with close reduction, three hematomas (0.4%:3/653), two adjacent segment diseases (0.9%:2/207), and one metal allergy (0.1%:1/653). In hip dislocation, the primary diagnoses included three cases of infected THA and 1 septic arthritis of the hip.

Aseptic loosening (type 2 failure) occurred in of 20/653 cases (3.0%). The primary diseases caused by aseptic loosening included 15 cases of tumors in the extremities, two infected TKA, and one case each of spinal tumor, pyogenic spondylitis, and arthritis. Revision of the iodine-coated implants was performed in all cases, with good results.

Breakage of the implants and peri-implant fractures (type 3 failure) occurred in 59/653 cases (9.0%). Breakage of the implants included 40 implant fractures (6.1%) and three broken bushes of tumor prostheses. Breakage of the pedicle screw was the most frequent implant failure, occurred in 29/653 (4.4%) patients. Other implant breakages included 10 broken plates using recycled bone fixation for tumor surgery (1.5%:10/653) and one megaprosthesis stem fracture (0.1%:1/653). The peri-implant fracture included 14 frozen bones for reconstruction after tumor excision (2.1%:14/653) and one case each of infected TKA and pseudoarthrosis. All patients underwent revision surgery, with good results.

Local tumor progression (type 5 failure) occurred in 8.7% (26/297) patients with tumors. The sites of tumor recurrence included 13 extremities and 13 spines.

## Infection (type 4 failure)

**Fig. 1:**
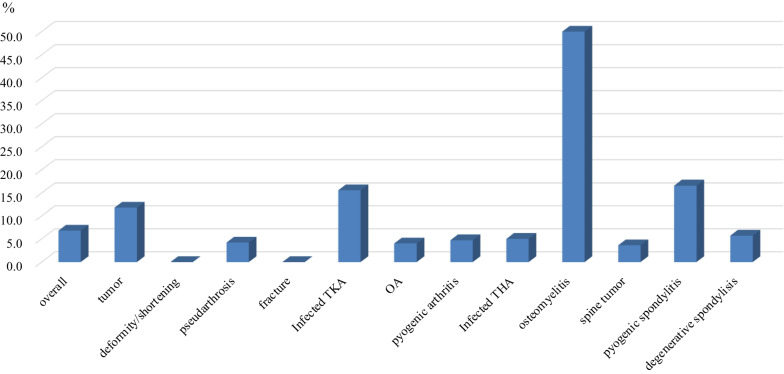
Overall infection rate for each disease

The total number of infected cases was 45/653 cases (6.8%). The infection rate of limb osteomyelitis was highest at 50% (3/6), followed by purulent spondylitis at 16.6% (6/36), infected TKA at 15.6% (5/32), extremity/pelvic tumor at 11.8% (19/161), spinal degeneration at 5.7% (2/35), infected THA at 5.0% (1/20), pyogenic arthritis at 4.7% (1/21), pseudarthrosis at 4.2% (2/47), osteoarthritis at 4.0% (1/25), spinal tumors at 3.6% (5/136), and deformity/shortening fractures at 0% (Fig. 1). The mean time to the onset of infection after implantation was 9.1 months (range, 1–19 months). The pathogenic bacteria were unknown in 20 cases, methicillin-resistant Staphylococcus aureus (MRSA) in 8 cases, Staphylococcus epidermidis in 6 cases, Staphylococcus aureus in 3 cases, Pseudomonas aeruginosa in 2 cases, Escherichia coli in 2 cases, one case each of methicillin-resistant Staphylococcus epidermidis (MRSE), Streptococcus, Corynebacterium striatum, and enterococcus faecalis.

**Fig. 2:**
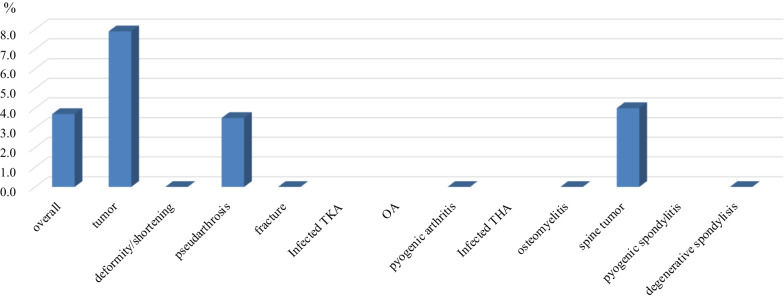
Infection rate of each disease in preventive cases

The total infection rate of cases used for prophylactic purposes was 3.7% (18/477), and most of these infections occurred in tumor cases (limb/pelvic tumor, 7.9%, 11/138; spinal tumor, 4%, 5/125) (Fig. 2).

**Fig. 3:**
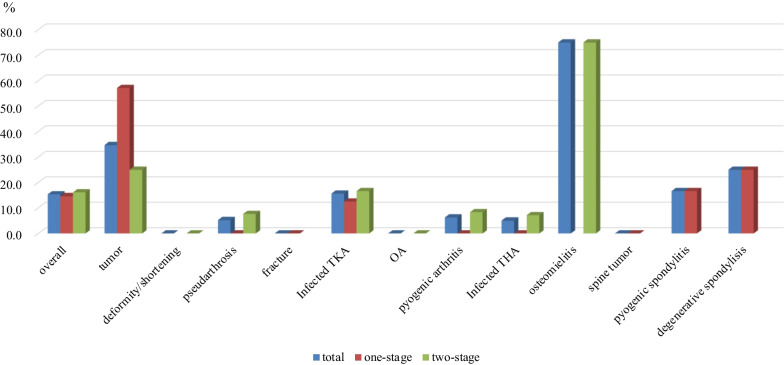
Infection rate of each disease in treated cases

The total infection rate of cases aimed at treating infection was 15.3% (27/176), and there was no difference between one-stage replacement (14.6%, 13/89) and two-stage replacement (16.0%, 14/87). Osteomyelitis of the extremities was highest at 75% (3/4), followed by limb/pelvic tumors at 34.7% (8/23), purulent spondylitis at 16.6% (6/36), and infected TKA at 15.6% (5/23) (Fig. 3). There were 11 cases of treatment for postoperative spinal tumor infection, and the re-infection rate was 0% using iodine-coated instruments.

Ultimately, all 45 infected patients were cured. Three patients (6.6%) were cured with antibiotics alone, and 16 (35.5%) were cured with debridement, antibiotics, and implant retention (DAIR). Six cases (13.3%) were cured with partial replacement, and three cases (6.6%) were cured with additional surgery. One case (2.2%) was cured by partial removal of the implant, five cases (11.1%) were cured by total removal of the implant, and 11 cases (24.4%) were cured with additional surgery. Additional surgeries included amputation in three cases (two tumors and one pyogenic osteomyelitis) (Table 3).

**Table 3:** Treatment of infection

	No. of cases	Relative incidence	Absolute risk	Mean time to infection (months)	Treatment of infection						
					Antibiotics	DAIR	Partial revision	Partial revision, additional surgery	Partial removal	Total removal	Total removal, additional surgery (Amputation)
Overall	45	26.2% (45/172)	6.8% (45/653)	11.9	3	16	6	3	1	5	11 (3)
Prevention	18	10.5% (18/172)	3.7% (18/477)	10.2	1	5	3	1	0	4	4 (1)
Treatment	27	15.7% (27/172)	15.3% (27/176)	13.1	2	11	3	2	1	1	7 (2)

## Discussion

In this prospective clinical study, iodine-supported implants were found to be useful for the prevention and treatment of postoperative infections in compromised hosts.

Several factors may contribute to implant failure: soft tissue defects for tumor cases and poor soft tissue condition in revision cases leading to instability [3, 4] bone quality and cement technique that may contribute to loosening and prosthetic or peri-prosthetic fracture [3]; chemotherapy and radiotherapy, large bone defects and prostheses, increasing surgery time (> 2.5 h), increased patient body mass index, lower preoperative hemoglobin or albumin levels, and postoperative hematoma contribute to a higher risk of infection [3, 5].

In this study, four patients (9%) had hip dislocation as type 1 failures. In all the patients, iodine-coated implants were used for therapeutic purposes. Garbuz et al. [6] reported that the dislocation rate in revision THA using a 32-mm head was 8.7%, and hip dislocation was independent of the iodine coating.

Aseptic loosening (type 2) accounted for 3% of cases in this study. Tumors accounted for the majority of cases. The incidence of aseptic loosening failure of tumor prostheses in the literature is 4.7–10% [3]. In comparison, the aseptic loosening rate of iodine-coated implants (3.0%) was satisfactory.

In this study, the structural failure (type 3) rate was 9.0%, including an implant fracture rate of 6.1%. Spinal instrument fractures were most common (4.4%). The mechanical failure of spinal instruments is reported to be 2.0–10% [7, 8]. On the other hand, the mechanical failure of a magaprosthesis is reported to be 11.7% [9]. In the study by Qu et al. [10], the rate of fixation failure for tumor-bearing bone was 7.4%. The implant fracture rate in the current study was 1.5%, which is better than that reported in these studies. Peri-implant fractures are also associated with structural failures. Previous reports have reported periprosthetic fractures after THA and TKA in 0–18% and 0.3–2.5% of patients, respectively [11, 12]. In this study, there was only one case of periprosthetic fracture, which was very rare. In contrast, most peri-implant fractures were recycled bone fractures in patients with tumors. In a previous report, Paholpak et al. [13] reported that fractures of frozen bone using liquid nitrogen occurred in 8% of patients. In comparison, 2.1% of patients in our study had such fractures which indicates a very low rate. We believe that the small number of structural failures is because iodine-coated implants have high bone affinity and good fixation.

Infection (type 4) is the worst and most frequent cause of failure [9, 14–16]. In this study, the final infection rate using iodine-coated implants was 6.8% (3.7% for prevention and 15.3% for treatment) in immunocompromised hosts and in postoperatively infected cases. The incidence of SSI using spinal instruments is reported to be 4.4–21.9% in the literature [15, 16]. Pala et al. [9] reported an infection rate of 6.9% when silver-coated titanium implants were used to prevent infection, which was similar to the infection rate when iodine coating was used. On the other hand, according to Fiore et al. [17], when a silver-coated maegaprosthesis was used for infection prevention and treatment, the overall infection rate was 17.6% (primary: 9.2%, revision: 13.7%). The results of this study indicate that iodine-coated implants are superior to non-coated implants. Because many of the infected cases in this study were refractory bacterial infections (osteomyelitis, pyogenic spondylitis, infected THA, and infected TKA), re-infections were likely to occur. According to previous reports, staphylococci are the most common cause of postoperative infections, especially MRSA and MRSE [18]. In this study, MRSA was also detected as the most common pathogen. In revision surgery, two-stage revision surgery is usually recommended [5, 19]; however, in this study, there was no significant difference in the re-infection rate between one-stage and two-stage replacement (14.6% vs. 16.0%, respectively). These rates were lower than those reported by Nucci et al. [19] (45.5% vs. 27.3%, respectively). Pala et al. [9] reported a mean time to the onset of infection of 25 months. In the current study, the mean time to the onset of infection was 9.1 months, which tended to be short. Because the patients in this study were immunocompromised hosts and patients with postoperative infections, this may have resulted in a reduction in the time of infection onset. All 45 infected cases were eventually cured, but limb amputation was performed in three cases (6.6%, 3/45). In previous reports, post-infectious amputations ranged from 23.5 to 87% [20]. In comparison, treatment with iodine-coated implants greatly reduced amputation. Furthermore, a one-stage replacement surgery for postoperative spinal infection did not result in infection. This result has the potential to alter conventional wisdom.

Tumor progression (type 5) failure occurred in 4.3–4.8% of cases in the literature [3, 14]. In the current study, type 5 failure occurred in 26/297 (8.7%) patients, which tended to be slightly higher than previously reported. This is because many metastatic tumors were treated.

The present study had several limitations. First, there was heterogeneity in the primary diseases. Second, various types of implants were used, such as plates, prostheses, and spinal instruments. Third, this study was a comparison with historical controls and was not a randomized controlled trial. However, the positive effect of iodine-supported implants in preventing and treating postoperative infection is encouraging and warrants further study.

## Conclusions

The five modes of failure of the iodine-supported implant were satisfactory compared with those of previous reports. In particular, because the infection rate of iodine-coated implants used for compromised hosts is low compared with those of other methods, postoperative infection is more easily controlled. It can also be considered highly effective for spinal infections that require one-stage revision surgery.

## Data Availability

The datasets used during the current study are available from the corresponding author on reasonable request.

## Abbreviations

DAIR: Debridement, antibiotics, and implant retention
MRSA: Methicillin-resistant Staphylococcus aureus
MRSE: Methicillin-resistant Staphylococcus epidermidis
SSI: Surgical site infection
THA: Total hip arthroplasty
TKA: Total knee arthroplasty
